# How are we measuring health-related quality of life in patients with a Barrett Esophagus? A systematic review on patient-reported outcome measurements

**DOI:** 10.1007/s11136-021-03009-7

**Published:** 2021-11-08

**Authors:** Mirjam C. M. van der Ende-van Loon, A. Stoker, P. T. Nieuwkerk, W. L. Curvers, E. J. Schoon

**Affiliations:** 1 Department of Gastroenterology and Hepatology, Catharina Hospital, Michelangelolaan 2, 5623 EJ Eindhoven, The Netherlands; 2 Department of Medical Psychology, Academic Medical Center, Amsterdam, The Netherlands; 3 GROW: School of Oncology and Developmental Biology, Maastricht University, Maastricht, The Netherlands

**Keywords:** Barrett Esophagus, Quality of life, Patient-reported outcome measures, PROM

## Abstract

**Purpose:**

Barrett esophagus (BE) is associated with a significant decrease of health-related quality of life (HRQoL). Too often, patient-reported outcome measures (PROMs) are applied without considering what they measure and for which purposes they are suitable. With this systematic review, we provide researchers and physicians with an overview of all the instruments previously used for measuring HRQoL in BE patients and which PROMs are most appropriate from the patient’s perspective.

**Methods:**

A comprehensive search was performed to identify all PROMs used for measuring HRQoL in BE patients, to identify factors influencing HRQoL according to BE patients, and to evaluate each PROM from a patients’ perspective.

**Results:**

Among the 27 studies, a total of 32 different HRQoL instruments were identified. None of these instruments were designed or validated for use in BE patients. Four qualitative studies were identified exploring factors influencing HRQoL in the perceptions of BE patients. These factors included fear of cancer, anxiety, trust in physician, sense of control, uncertainty, worry, burden of endoscopy, knowledge and understanding, gastrointestinal symptoms, sleeping difficulties, diet and lifestyle, use of medication, and support of family and friends.

**Conclusion:**

None of the quantitative studies measuring HRQoL in BE patients sufficiently reflected the perceptions of HRQoL in BE patients. Only gastrointestinal symptoms and anxiety were addressed in the majority of the studies. For the selection of PROMs, we encourage physicians and researchers measuring HRQoL to choose their PROMs from a patient perspective and not strictly based on health professionals’ definitions of what is relevant.

## Introduction

Barrett’s esophagus (BE) is a premalignant condition involving metaplastic transformation of the lower esophageal lining from squamous to intestinal epithelium, due to gastroesophageal reflux disease (GERD) [1, 2]. BE is associated with an increased risk of an esophageal adenocarcinoma (EAC). The relative risk of EAC in patients with non-dysplastic BE is 30–125 times higher compared to the general population. Patients therefore undergo regular endoscopic surveillance for early detection of malignant transformation. Although early detection may lead to improved survival, the absolute risk for malignant transformation is low (approximately 0.3–0.5% per year) [3, 4] and the efficacy of surveillance and the influence of BE on life expectancy are still questioned [4–7]. The effect of endoscopic surveillance programs on patient’s perspective and quality of life should, therefore, not be neglected [8].

BE is associated with a significant decrease of health-related quality of life (HRQoL), measured with both generic and disease-targeted instruments [9]. In addition, patients with BE are at risk for psychological consequences such as depression, anxiety, and stress. These negative effects of BE on HRQoL and psychological health may be related to patients’ perception of the risk of developing EAC [9]. HRQol is generally considered to encompass patients’ physical, psychological, and social functioning, which can be affected by both the disease and treatment [10].

Nowadays, there is an increased awareness in international health care policy on the importance of measuring quality of care. Patient-reported outcomes (PRO) are an important instrument for measuring quality of care, enabling improvement and transparency in health care. The choice of what to measure (PRO) and how to measure is a complicated but important process. Too often, patient-reported outcome measurements (PROMs) are applied without considering what they should measure and for which purposes they are suitable. There is a rapid increase of questionnaires to choose from, however, it is often not clear which one is the best given its purpose. Currently, there is no BE-specific PROM available.

In this systematic review, we will identify all PROMs used for measuring HRQoL in BE patients, identify factors influencing HRQoL according to BE patients, and evaluate each PROM from a patient’s perspective. This systematic review is part of a research project on the development of a person-centered measurement tool, measuring HRQoL in BE patients.

## Materials and methods

This systematic review was performed in accordance with the preferred reporting items for systematic reviews and meta-analyses (PRISMA) statement [11].

### Literature search

**Fig. 1:**
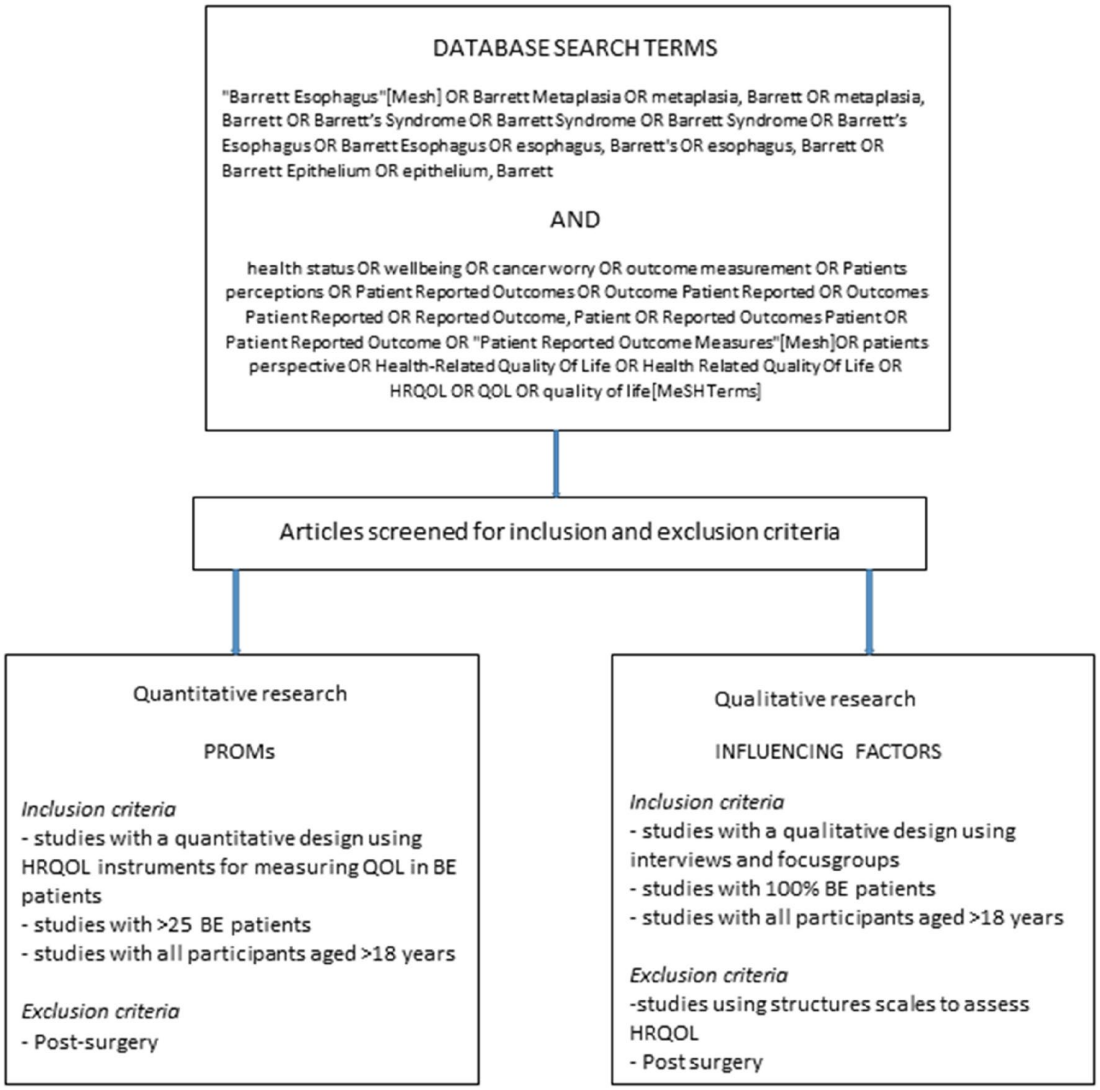
Database search in- and exclusion criteria

Two independent researchers (MvdE and AS) independently conducted a systematic search from inception to February 1, 2021 in the following electronic databases: Pubmed, EMBASE, CINAHL, and PsycINFO. To search the databases, we used medical subject headings (MeSH) and free-text words (Fig. 1). We additionally carried out reference and citation searches of all included articles and relevant review articles.

### Inclusion and exclusion criteria

Studies were included when they were written in English and included only patients over 18 years old. Each article was judged against two sets of inclusion criteria (Fig. 1).

1. Studies using HRQoL PROMs were included when they met the following criteria:

- Using one or more PROMs for assessing HRQoL in BE patients. A PROM was defined as any self-administered QOL instrument assessing one of the three core domains described by the World Health Association: physical, social, and psychological well-being [12].
- Measuring HRQoL in patients with a study population containing more than 25% BE patients. With this criterion, we aimed to ensure that the authors chose their PROMs from a perspective of the BE population. Subsequently, we used a criterion of inclusion of n>25 to guarantee an acceptable quality of the included articles with a quantitative approach.

Studies with primarily post-surgery measurements were excluded.

(2) Studies on influencing factors were included when they met the following criteria:

- Using a qualitative methodology (e.g., focus groups or in-depth interviews).
- Studies including only BE patients.

### Data extraction and analysis

1. Identification of PROMs

The details of all included studies (e.g., aim, sample sizes, study objectives, the level of evidence according to the Oxford Centre for Evidence-Based Medicine (OCEBM) criteria [13], and the PROMs used for measuring HRQoL) were reported in a summary table. Subsequently, it was determined whether a validation in the BE population was described in the reference literature of the included articles. Objectives and domains of each PROM were obtained. PROMs measuring perceived cancer risk, time trade-off, and standard gamble scores were not used for analyses.

(2) Identification of influencing factors according to BE patients

To identify factors influencing HRQoL according to BE patients, quality assessment was independently conducted by two researchers (MvdE and AS) using the Critical Appraisal Skills Programme (CASP) criteria; a 10-item checklist designed for use in the appraisal of qualitative research studies [14]. In addition, factors were evaluated according to their relevance. To evaluate intra-rater and inter-rater reliability in the factors extracted from the literature review, two reviewers (MvdE and AS) each independently extracted a list of potential factors from the articles included. The two lists were compared, and differences resolved by consensus. All influencing factors identified were categorized into domains according to the patient-reported outcomes measurement information system (PROMIS) Adult Self-Reported Health model [15].

(3) Evaluation of each PROM

Finally, each PROM was evaluated in terms of its ability to capture factors important to BE patients. For each factor, it was examined whether this was measured with an item of the PROM. A distinction was made between addressing a factor directly or indirectly in an item of the questionnaire. For example, when a questionnaire inquired about pain in general, the factor epigastric pain was considered to be measured indirectly.

## Results

**Fig. 2:**
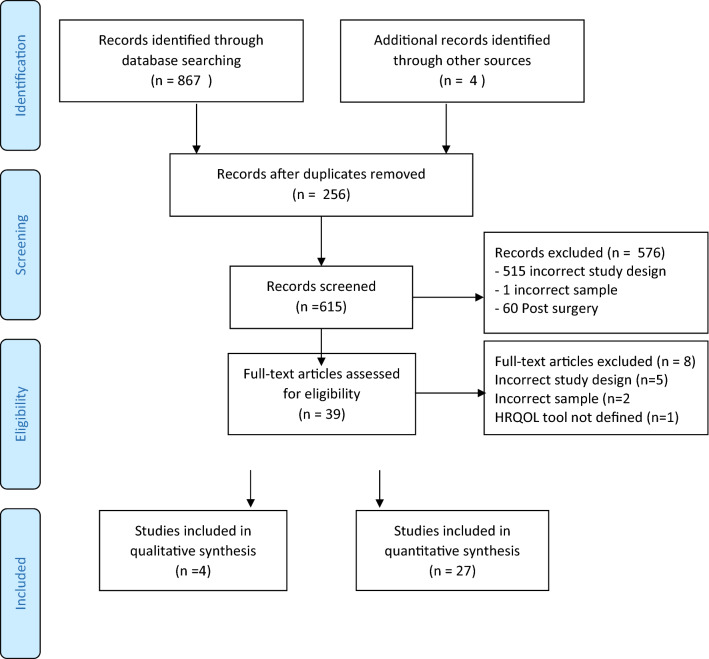
PRISMA 2009 Flow diagram

The literature search identified 402 articles. Twenty-seven articles met the inclusion criteria for HRQoL PROMs, after manual review of the full texts, and were included for analysis. Four qualitative studies that met the criteria for influencing factors were included (Fig. 2).

### Identification of PROMs

Among the 27 studies [16–42], 32 different PROMs (Table 1) were identified. A total of nine studies [16, 21, 22, 31, 34, 35, 40–42] used PROMs that were not formally validated.

**Table 1:** PROMs used for measuring HRQoL in Barrett esophagus patients

Abbreviation type		Objective	Domains covered
SF-36	Generic	Measuring HRQoL of individuals with several chronic health conditions	36-questions on physical functioning, physical role, pain, general health, vitality, social function, emotional role and mental health
SF-12	Generic	Measuring HRQoL of individuals with several chronic health conditions with substantially fewer questions than the SF-36	12-Questions on physical functioning, role functioning, social functioning, mental health, health perceptions, pain
EQ-5D (3L or 5L)*	Generic	A simple, generic measure of health for clinical and economic appraisal	5-Items on mobility, self-care, usual activity, pain/discomfort, and anxiety/depression and a visual analogue scale on self-rated health
PROMIS-10	Generic	Measurements of symptoms, functioning, and healthcare-related quality of life (HRQoL) for a wide variety of chronic diseases and conditions	10-Questions on overall physical health, mental health, social health, pain, fatigue, and overall perceived quality of life
LASA	Generic	General measures of global QOL dimensional constructs in numerous settings	5-Questions on physical well-being, emotional well-being, spiritual well-being, intellectual well-being, and overall QOL
WHOQOL-BREF	Generic	Assess the individual’s perceptions in the context of their culture and value systems, and their personal goals, standards, and concerns	26-Questions on global items, physical health, psychological health, social relationships, environment QOL
EORTC-QLQC30	Cancer specific	Assessing the HRQoL of cancer patients participating in international clinical trials	30-Questions on functional scales, symptom scales, global health status/QoL scale, and a number of single items assessing additional symptoms commonly reported by cancer patients and perceived financial impact of the disease
EORTC-QLU-C10D	Cancer specific	Developed to capture cancer patients’ QoL and to relate it to survival time and costs of treatment in health economic studies	10-Items on physical functioning, role functioning, social functioning, emotional functioning, pain, fatigue, sleep, appetite, nausea, bowel problems
GERD-Q	Disease symptoms specific	Determine the presence or absence of symptoms of GERD in the general population	6-Questions on symptoms of GERD
BSI-18	Disease symptoms specific	Assessment of psychological distress	18-Questions on somatization, anxiety, and depression
GSRS	Disease symptoms specific	A clinical rating scale for gastrointestinal symptoms in patients with irritable bowel syndrome and peptic ulcer disease	15-Qestions on reflux, abdominal pain, indigestion, diarrhea, and constipation
GIQLI	Disease symptoms specific	Assess QoL specific for the gastrointestinal tract	36-Questions on GI symptoms, emotion, physical function, social function, and medical treatment
SCL-90	Disease symptoms specific	Evaluate a broad range of psychological problems and symptoms of psychopathology	90-Items on somatization, obsessive compulsive disorder, interpersonal sensitivity, depression, anxiety, hostility, phobic anxiety, paranoid ideation, psychoticism
GERD-HRQL	Disease symptoms specific	Measure symptomatic change as a result of medical or surgical treatment of GERD	16-Questions on measuring symptom severity in GERD
QOLRAD	Disease symptoms specific	Monitor changes in HRQOL in patients suffering from heartburn and dyspepsia	25-Questions on emotional distress, sleep disturbances, food/drink problems, physical/social functioning, vitality
RDQ	Disease symptoms specific	Assess the frequency and severity of heartburn, regurgitation, and dyspeptic complaints and to facilitate the diagnosis of GERD in primary care	12-Items on regurgitation, heartburn, and dyspepsia
BE QOL	Disease symptoms specific	Not defined	10-Questions on esophagostomy worry, adenocarcinoma worry, esophagus general worry, depression, daily QoL, amount of stress, difficulty to sleep, work or family life negatively impacted and worry dying due to esophagus
The ogilvie grading scale	Disease symptoms specific	To determine level of dysphagia	5-Items on dysphagia
QLQ-OG25	Cancer symptom specific	Assess QOL in patients with esophageal or gastric cancer and esophagogastric junction carcinoma	25-Questions on dysphagia, eating restrictions, reflux, odynophagia, pain, and anxiety
EORTC-QLQ OES18	Cancer symptom specific	Assess QOL in patients with esophageal cancer	18-Questions on esophageal functional, symptomatic scales, and the global QoL
TPS	Trust in physician	Assess each patient’s interpersonal trust in his primary care physician within the context of the management of chronic disease	11-Items on trust in physician
IES	Endoscopic burden	Assess current subjective distress for any life event	15-Items on episodes of intrusion, episodes of avoidance
DIS	Endoscopic burden	Measure of avoidance of and difficulty in tolerating somatic sensations	7-Items on ability to tolerate discomfort and pain, and avoidance of physical discomfort
PSQI	Sleeping difficulties	Assess sleep quality over a 1-month time interval	19-Items on subjective sleep quality, sleep latency, sleep duration, habitual sleep efficiency, sleep disturbances, use of sleeping medication, and daytime dysfunction
Berlin-Q	Sleeping difficulties	Identifying patients with sleep apnea in primary care setting	10-Questions on snoring behavior, wake time sleepiness or fatigue, obesity, hypertension
HADS	Anxiety and depression	Measure symptoms of anxiety and depression	14-Items on anxiety and depression
B-IPQ	Illness perceptions	Assess cognitive and emotional representations of illness	8-Questions on cognitive illness, emotional perceptions, illness comprehensibility. And an open-ended response with three most important self-perceived causal factors of their illness
WOCS	Fear of cancer	Undefined	4-Questions on esophageal cancer in particular
CWS	Fear of cancer	Measure cancer-specific worry and impact of worry on daily functioning	8-Questions on worry and impact of worry on daily functioning

*RDQ* The reflux disease questionnaire, *QOLRAD* Quality of life in reflux and dyspepsia, *GERD-HRQL* The gastroesophageal reflux disease-health-related quality of life, *EQ-5D* EuroQOL-5D, *GERD-Q* Gastroesophageal reflux disease-questionnaire, *EORTC-QLQ-OES1* The European Organization for Research and Treatment of Cancer-Quality of life questionnaire-Oesophageal cancer module, *EORTC-QLQ-C30* The European Organization for Research and Treatment of Cancer-Quality of life questionnaire, *HADS* Hospital anxiety and depression scale, *SF-16* The 16-item short form health survey questionnaire, *SF-12* The 12-item short form health survey questionnaire, *SF-6D* Short form-6 dimension, *PROMIS-10* Patient-reported outcomes measurement information systems, *LASA* Linear analog self-assessment, *WHOQOL-BREF* World health organization quality of life instruments, *EORTC-QLU-C10D* European Organization for Research and Treatment of Cancer-Quality of life questionnaire-Core 10, *BSI-18* Brief symptom inventory, *GSRS* Gastrointestinal symptom rating scale, *GIQLI* Gastrointestinal quality of life index, *SCL-90* The symptom checklist-90-revised, *QLQ-OG25* EORTC quality of life questionnaire–Oesophago-gastric module, *TPS* Trust in physician scale, *IES* The impact of event scale, *DIS* The discomfort intolerance scale, PSQI, *Berlin-Q* Berlin questionnaire, *B-IPQ* Brief-Illness perception scale, *CWS* Cancer worry scale, *WOCS* Worry of cancer scale

*The EQ−5D−5L differs from the EQ−5D−3L on the following points: (1) The number of levels of perceived problems per dimension was changed from 3 to 5. The most severe label for the mobility dimension was changed from “confined to bed” to “unable to walk about," and the instructions for the EQ VAS task were simplified

The study of Shaheen et al. [31] used a disease-specific BE questionnaire. However, to our knowledge, this specific BE questionnaire has not been properly validated.

An average of 3 (range 1–5) PROMs per study were used. Table 2 demonstrates a summary of sample and design characteristics of studies reporting HRQoL in BE patients. The mean number of PROMs used per study did not change over the years. Three Level 2 studies were found using PROMs in a RCT design. The majority (87.9%) were Level 3 studies per OCEBM criteria [13].

**Table 2:** Study characteristics

Author, Year	Eloubeidi, 2000	Kulig, 2003	Gerson, 2005	Kruijshaar, 2006	Essink-Bot, 2007	Reddy, 2020	Gerson, 2007
Level of evidence	3	3	3	3	3	3	3
Analyse sample	NDBE = 88, GERD = 88	NDBE = 702, NERD = 2853 ERD = 2660	NDBE = 40, GERD = 118	NDBE = 180	NDBE = 180, NS = 214, EAC = 82	DBE/EAC ET = 239 DBE/EAC surgery = 153	NDBE/DBE = 60 GERD = 40
AIM	(1) To compare HRQL of patients with BE and patients with GERD who do not have BE; (2) to compare HRQL of GERD patients to that of normative data for the US general population; (3) to examine the impact of GERD symptom severity and frequency on HRQL in these patients	Describe the impact of GERD on the quality of life, to assess the changes in the QoL after 2 weeks of treatment with PPI and to define the factors that can predict these changes	To derive health state utilities for patients with chronic reflux symptoms who required daily medication for adequate symptom control	To explore the burden of upper gastro-intestinal endoscopy as perceived by patients	Analyze potential determinants of the perceived burden of upper GI endoscopy by comparing BE patients with two additional patient groups, i.e., patients with non-specific upper GI symptoms (NS) and patients with a recent diagnosis of cancer of the upper GI tract (CA)	Compare long-term HR-QOL associated with ET or esophagostomy among patients with HGD or T1a EAC	To determine whether time trade of values would differ in patients with BE when patients were asked to trade away potential risk of esophageal adenocarcinoma rather than chronic heartburn symptoms
Baseline characteristics	Age, race, gender, nicotine use, alcohol use, PPI use, Charlson index (comorbidities), psychosomatic symptom checklist	Age, gender education, marriage status, comorbidity, family history of GERD, nonsteroidal anti-inflammatory drug use, esophagitis, BMI	Age, gender, years of reflux, comorbidity, PPI use, 24-Kr potential of hydrogen test, and esophageal motility assessment	Age, gender, marital status, employment status, education, number of endoscopies, histology, reflux esophagitis, PPI use, general health	Age, gender, employment, civil status, education, sedation, hospital, endoscopy number	Age, gender, length BE diagnosis, histology, comorbidity	Age, gender, years of reflux, comorbidity, years on PPI, race, site of care, income
PROMs used	2	3	2	3	4	2	3
Validated PROMs	SF-36 GERD-Q	SF-36 QOLRAD RDQ	QOLRAD GSRS	EQ-5D-3L IES HADS	EQ-5D-3L IES HADS	EORTC-QLQ-C30 EORTC-QLQ-OES18	SF-36 QOLRAD RDQ
Non-validated questionnaires	N/A	N/A	N/A	Non-validated questions on disease symptoms with Likert scale	Non-validated questions on disease symptoms and endoscopic burden with Likert scale	N/A	N/A
Factors covered	7/18	8/18	7/18	3/18	3/18	7/18	8/18

Author, Year	Lippmann, 2009	Cooper, 2009	Miller, 2010	Rosmolen, 2010	Shaheen, 2010		Schembre, 2010
Level of evidence	3	3	3	3	2		3
Analyse sample	NDBE = 168, GERD = 361	NDBE = 151	NDBE/DBE = 489, EAC = 212	DBE/EAC ET = 81 EAC surgery = 33	DBE = 127		DBE = 40
AIM	To isolate any decrease in HRQoL associated with Barrett’s esophagus by comparing BE patients to GERD patients with similar GERD symptom severity, and to measure any additional psychological distress that may be associated with BE, which could potentially be attributed to cancer risk. Additionally, we sought to determine whether any differences were present in quality of life based on gender and presence of erosive disease	Examine the experience of patients undergoing endoscopic surveillance for BO, their levels of anxiety and depression, and quality of life and how the relationship with their physicians influences these factors	To quantify the association of marital status and changes in QOL over time in patients with EC and patients with BE	To explore QOL, fear of cancer recurrence, and anxiety in patients with a Barrett’s esophagus treated for HGD or early cancer in the past, by comparing these outcomes between endoscopically and surgically treated patients	To evaluate QoL before and after endoscopic treatment of dysplastic BE with RFA		Attempt to better understand the relative impact of esophagestomy and ET on patients’ QOL after therapy and recovery are complete
Baseline characteristics	Age, gender race, alcohol use, tobacco use, anti- reflux surgery, BMI, medication, comorbidities, prior mental health status	Age, gender, number of gastroscopies, length BE	Age, gender, marriage status, histology, surgical treatment, chemotherapy	Age, gender, comorbidity. Endoscopy treatment: type of treatment, duration of the treatment, HGD/early cancer during follow-up. Surgically treated patients: type of surgical resection and reconstruction, length of hospital admission, complications, anastomotic stenosis, and histology of the resected specimen	Age, gender, race, BMI, Length of BE, histology, time since diagnosis of BE, time since diagnosis of dysplasia		Age, gender, American Society of Anesthesiologists score, BE length
PROMs used	4	3	1	4	1		2
Validated PROMs	SF-36 GIQLI SCL-90-R GERD-HRQL	SF-36 TIPS HADS	LASA	SF-36 EORTC-QLQ-C30 EORTC-QLQ-OES18 HADS			SF-36 GIQLI
Non-validated questionnaires	N/A	Non-validated question on trust Physician, fear of cancer and knowledge with and without Likert scale	N/A	WOCS	Eight non-validated questions with range scale		N/A
Factors covered	10/18	3/18	1/18	8/18	0/18		9/18

Author, Year	Crockett, 2012	Vela, 2013	Rosmolen, 2017	Chang, 2016	Lee, 2017	Baldaque-silva, 2017	Britton, 2020
Level of evidence	3	3	3	3	3	3	3
Analyse sample	NDBE = 235	NDBE = 63, GERD = 83 Control = 75	NDBE = 44, DBE/EAC ET = 42 DBE/EAC surgery = 21 Advanced EAC surgery = 19	NDBE = 84, control = 168	NDBE = 139	NDBE = 54	NDBE = 305 DBE = 48 GORD = 131 Colonic- polyp = 150 Control = 47
AIM	To identify predictors of over- or under-utilization of endoscopic surveillance including demographic factors, quality of life, healthcare numeracy, risk perception, and other health behaviors	(1) to compare the effect of GERD and BE on sleep quality and (2) to assess whether the association between sleep quality and GERD or its more severe form (i.e., BE) is independent of obstructive sleep apnea	Investigate the overall QOL and the fear of cancer recurrence at multiple time points and included larger cohorts of patients	Determine whether HRQOL of BE patients were worse than healthy referents in the ethnic Chinese population in Taiwan, adjusted for potential confounding factors	To investigate HRQoL in a Chinese population with BE	Determine the impact of upward titration of PPI on acid reflux, symptom scores, and histology, compared to clinically successful fundoplication	Assess HRQoL in patients with NDBE and endoscopically treated DBE compared with other common gastrointestinal disorders and healthy individuals
Baseline characteristics	Age, gender, race, site, education, income, employment, family history BE and EAC, insurance, duration of BE	Age, gender, race, smoking, BMI, recruitment source	Age, gender, comorbidity, type of treatment, treatment-related complications, treatment time, histology, recurrence during FU, additional treatment	Age, gender, BMI, comorbidity, marital status, education, employment, history of smoking and drinking	Age, BMI, Waist (cm), gender BE length, esophagocardiac junction, histology	Age, gender BMI, smoking, BE length	Age, gender, histology, employment, family history, career, smoking, PPI, anti-depressant, BE length, co-morbidities
PROMs used	2	3	4	1	3	1	4
Validated PROMs	SF-36 GERD-HRQL	GERD-Q PSQI BQ	SF-36 EORTC-QLQ-C30 EORTC-QLQ-OES18 HADS	WHOQOL-BREF	SF-12 RDQ HADS	GERD-HRQL	SF-36 GSRS CWS HADS
Non-validated questionnaire	Non-validated questions on disease symptoms, anxiety and worry with Likert scale	N/A	WOCS	N/A	N/A	N/A	N/A
Factors covered	7/18	6/18	8/18	3/18	7/18	7/18	7/18

Author, Year	Han, 2018	Ende-van Loon, 2018	Rosmolen, 2019	Balamu, 2019	Peerally, 2019	Schwameis, 2020	Hauge, 2020
Level of evidence	3	3	2	3	2	3	3
Analyse sample	NDBE/DBE = 193	NDBE = 158	NDBE = 49, DBE = 47	NDBE/DBE/EAC = 97	DBE/EAC = 76	DBE/EAC = 40	DBE/EAC = 86
AIM	(1) Measure QOL impairment among patients with BE referred for endoscopic eradication therapy; (2) identify factors associated with reduced QOL	To assess the EAC risk perceived by patients with NDBE in an endoscopic surveillance program and to associate these perceived EAC risks with illness perception and QoL	QOL and illness perceptions with confirmed low-grade dysplasia, comparing surveillance and ablation	Investigate HRQoL and health utility scores for common progression states in patients	Randomized pilot study of the 2 techniques comparing dysplasia clearance, BE eradication, recruitment, retention, and health economic analysis	To evaluate the workload associated with endotherapy, the frequency and type of recurrence, long-term QOL, and late oncologic outcomes in a group of patients that were followed for a minimum of 5 years by 1 treating physician	To evaluate the treatment of dysplasia and superficial esophageal cancer with endoscopic mucosal resection and/or radio frequency ablation and the post-procedural HRQL
Baseline characteristics	Age, gender, race, family history of BE and/or EAC, PPI use, duration of BE, length of BE (cm), histology, presence of Hiatus, Hernia Diaphragm, BMI	Age, gender BE diagnosis, marital status, education, employment status, comorbidity, cancer in friends or family	Age, gender length of BE, time since diagnosis of BE in years, time since diagnosis of dysplasia in years, PPI use, Number of comorbidities	Age, gender comorbidity, treatment history previous 12 months, smoking, race, born in Australia, smoking, comorbidities, treatment characteristics	Age, gender, BMI, BE length, histology	Age, gender, histology, no. Treatments, BE length, follow-up length	Age, gender, BE length, histology
PROMs used	4	3	4	5	3	3	3
Validated PROMs	PROMIS-10 Gerd-Q DIS BSI	SF-36 GERD-Q B-IPQ	SF-36 EORTC-QLQ-C30 EORTC-QLQ-OES18 B-IPQ	SF-6D SF-36 EQ-5D-5L EORTC-QLU-C10D EORTC-QLQ-C30	EQ-5D EORTC-QLQ-C30 EORTC-QLQ-OES18	SF-36 GIQLI	EORTC-QLQ-C30 QLQ-OG25 The Ogilvie grading scale
Non-validated questionnaire	N/A	N/A	N/A	N/A	Non-validated questions on disease symptoms	Non-validated questions on disease symptoms	N/A
Factors covered	7/18	9/18	7/18	3/18	8/18	9/18	7/18

*BE* Barrett esophagus, *NDBE* Non-dysplastic Barrett esophagus, *DBE* Dysplastic Barrett esophagus, *EAC* Esophageal adenocarcinoma, *GERD* Gastroesophageal reflux disease, *NERD* Nonerosive reflux disease, *NS* Non-specific upper GI symptoms, *ET* Endoscopic treatment, *HRQoL* Health-related quality of life, *QoL* Quality of life, *HGD* High-grade dysplasia, *BM* Body mass index, *PPI* Proton pomp inhibitor, *RDQ* The reflux disease questionnaire, *QOLRAD* Quality of life in reflux and dyspepsia, *GERD-HRQL* The gastroesophageal reflux disease-health-related quality of life, *EQ-5D* EuroQOL-5D, *GERD-Q* Gastroesophageal reflux disease-questionnaire, *EORTC-QLQ-OES18* The European Organization for Research and Treatment of Cancer-Quality of life questionnaire-Oesophageal cancer module, *EORTC-QLQ-C30* The European Organization for Research and Treatment of Cancer-Quality of life questionnaire, *HADS* Hospital anxiety and depression scale, *SF-16* The 16-item short form health survey questionnaire, *SF-12* The 12-item short form health survey questionnaire, *SF-6D* Short form-6 dimension, *PROMIS-10* Patient-reported outcomes measurement information systems, *LASA* Linear analog self-assessment, *WHOQOL-BREF* World health organization quality of life instruments, *EORTC-QLU-C10D* European Organization for Research and Treatment of Cancer-Quality of life questionnaire-Core 10, *BSI-18* Brief symptom inventory, *GSRS* Gastrointestinal symptom rating scale, *GIQLI* Gastrointestinal quality of life index, *SCL-90* The symptom checklist-90-revised, *QLQ-OG25* EORTC quality of life questionnaire-Oesophago-gastric module, *TPS* Trust in physician scale, *IES* The impact of event scale, *DIS* The discomfort intolerance scale, PSQI, *Berlin-Q* Berlin questionnaire, *B-IPQ* Brief-Illness perception scale, *CWS* Cancer worry scale, *WOCS* Worry of cancer scale

Seven different PROMs were used for measuring generic HRQoL (SF-36, SF-12, SF-6D, WHOQOL-BREF, LASA, PROMIS-10, and the EQ-5D for measuring health utility). Two disease-specific PROMs assessed the generic aspects of QOL in cancer patients (EORTC-QLQC30 and QLU-C10D). Fourteen different disease-specific PROMs were used, measuring symptoms related to BE (GERD-Q, GERD-HRQL, BSI, GSRS, GIQLI, SCL-90, QOLRAD, RDQ, EORTC-QLQOES18, QLQ-OG25, the EORTC-QLQ OES, QLQ-OG25 and five different non-validated questionnaires) [16, 34, 35, 40, 41]. Cancer worry was measured with the WOCS, CWS, and a non-validated questionnaire [42].

**Fig. 3:**
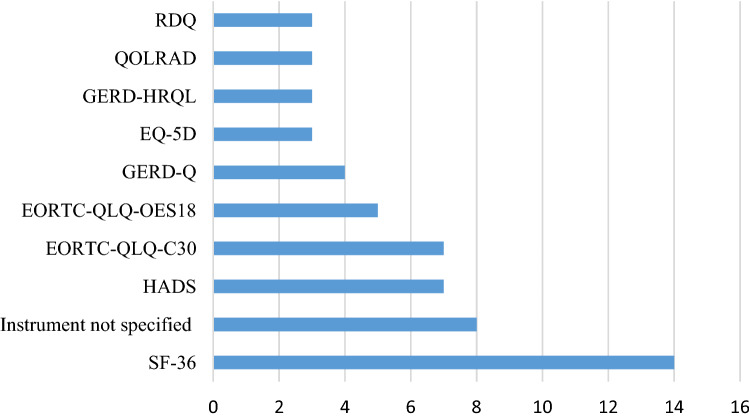
Top 10 most frequently reported PROMs

Two PROMs measured sleeping difficulties (PSQI, BQ). Endoscopic burden was measured with three different PROMs (IES, DIS, and a non-validated Likert scale questionnaire [41]). An additional number of PROMs were identified, measuring trust in physician using the trust in physician scale (TIPS), anxiety and depression (HADS and a non-validated Likert questionnaire) [34], illness perceptions (B-IPQ), knowledge with non-validated questionnaire [42], and trust in the endoscopy with a non-validated Likert questionnaire [42]. The 10 most frequently cited PROMs are illustrated in Fig. 3. All studies except four [24, 26, 31, 36] used some form of a generic PROM for measuring HRQoL. The SF-36 was utilized most often, respectively, in 51.8% of the studies. Symptoms related to BE were measured in 85.2% of studies. The EORTC-QLQ-OES18, GERD-Q, QOLRAD, RDQ, and GERD-HRQL were most frequently used to measure reflux symptoms. Non-validated questionnaires were used in 30% of all included studies.

### Identification of influencing factors according to BE patients

**Fig. 4:**
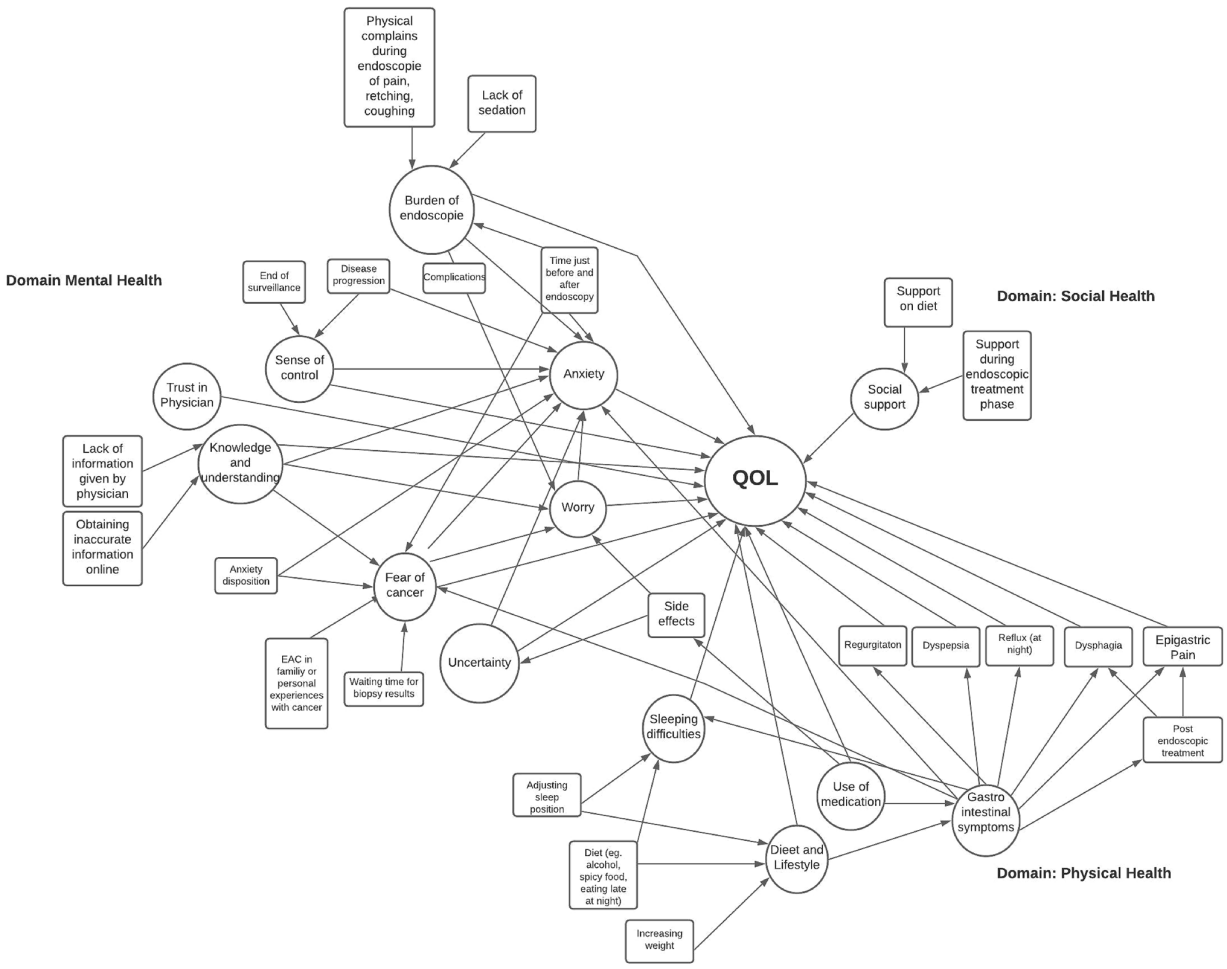
Conceptuel framework

Four studies with a qualitative design were identified: one study used a focus group design and three used patient interviews [43–46]. The study characteristics and quality scores are demonstrated in Table 3. Studies were published between 2011 and 2020 and were conducted in the UK (*n* = 2), USA (*n* = 1), and the Netherlands (*n* = 1). All studies showed a minimal quality score of 7/10 according to CASP [14]. Within these studies, the following factors related to HRQoL according to BE patients were identified, namely fear of cancer, anxiety, trust in physicians, sense of control, uncertainty, worry, burden of endoscopy, knowledge and understanding, gastrointestinal (GI) symptoms (e.g., reflux or heartburn, regurgitation, dyspepsia, dysphagia, epigastric pain), sleeping difficulties, diet and lifestyle, use of medication, and support of family and friends. These factors were allocated into domains and displayed in a conceptual framework (see Fig. 4).

**Table 3:** Summary list of domains and associated factors influencing HRQoL

Author, Year, Country	Ende-van Loon, 2020, NL		Britton, 2018, UK	Arney, 2014, USA	Griffiths, 2011, UK	
Aim	To assess the factors influencing HRQOL according to NDBE and DBE patients		To identify and explore factors impacting BO patients’ health-related quality of life, follow-up needs and views on new models of follow-up care	To identify elements of the EGD experience that frame patients’ memories and overall perceptions of surveillance		To explore patients’ views and perspectives on their experience of living with Barrett’s columnar-lined oesophagus (CLO) and being part of an endoscopic surveillance program
Method	Focus group		Exploratory qualitative approach was adopted using semi-structured, in-depth, one-to-one interviews	structured, in-depth, qualitative interviews	Qualitative semistructured interviews	
Sample	NDBE = 16 DBE/EAC ET = 17		NDBE = 20	NDBE/DBE = 20	NDBE = 22	
Quality score	10/10		10/10	8/10	7/10	
1. Mental health						
Fear of cancer	✓		✓	✓	✓	
Anxiety	✓		✓		✓	
Trust in physician	✓		✓		✓	
Sense of control	✓		✓	✓	✓	
Uncertainty	✓		✓	✓	✓	
Worry	✓		✓		✓	
Burden of endoscopy	✓		✓		✓	
Knowledge and understanding	✓		✓	✓	✓	
2. Physical health						
Gastrointestinal symptoms	✓		✓	✓	✓	
Reflux	✓			✓		
Regurgitation	✓					
Dyspepsia	✓					
Dysphagia	✓					
Epigastric pain	✓					
Sleeping difficulties	✓					
Diet/lifestyle	✓					
Use of medication	✓		✓	✓		
3. Social health						
Support of family and friends	✓					

*NL* Netherlands, *UK* United Kingdom, *USA* United States of America, *HRQOL* Health-related quality of life, *BE* Barrett esophagus, *NDBE* Non-dysplastic Barrett esophagus, *DBE* Dysplastic Barrett esophagus, *EAC* Esophageal adenocarcinoma

*Quality score using the CASP criteria; a 10−item checklist designed for use in the appraisal of qualitative research studies (CASP)16

### Coverage of factors in HRQOL PROMs relevant to patients

None of the 27 identified PROMs covered all factors important to BE patients (Table 4). Generic PROMs were used in 77.8% of all studies, and only a small number of factors were indirectly addressed. For instance, the commonly used SF 36 and SF12 contained items indirectly addressing anxiety and items on pain in general. The EQ-5D, PROMIS 10, LASA, WHOQOL-BREF had additional items on anxiety, and the EORTC-QLQC30 on worry.

**Table 4:** PROMs and the coverage of factors important to patients with BE

	Fear of cancer	Anxiety	Trust in physician	Sense of control	Uncer-tainty	Worry	Burden of endoscopy	Knowledge and understanding	Reflux/heartburn		Regurgitation		Dyspepsia		Dysphagia	Epi-gastric pain	Sleeping difficulties	Diet/lifestyle	Use of medication	Social Support	Total factors ✓ (±)
GIQLI		✓								✓		✓	✓	✓		✓	✓		✓	✓	9
GERD-HRQL										✓		✓	✓	✓		✓	✓	±	✓		7 (1)
GERD-Q										✓		✓	✓			✓	✓		✓		6
QLQ-OG25		✓				✓				✓			✓	✓		✓					6
QOLRAD		✓			±	✓				✓		✓					✓	±			5 (+ 2)
RDQ										✓		✓	✓	✓		✓					5
EORTC-QLQ OES18										✓		✓		✓		✓		✓			5
GSRS										✓		✓	✓			✓					4
WHOQOL-BREF		✓														±	✓		±	✓	3 (2)
SCL-90		✓				✓							±				✓				3 (1)
B-IPQ		±		✓		✓		✓													3 (1)
EORTC-QLQC30						✓										±	✓		±		2 (2)
CWS	✓					✓															2
HADS		✓				✓															2
EORTC QLU-C10D																±	✓		±		1 (2)
PROMIS-10		✓														±				±	1 (2)
EQ-5D		✓														±					1 (1)
IES							±										✓				1 (1)
BSI-18		✓																			1
TPS			✓																		1
LASA		✓																			1
PSQI																	✓				1
Berlin-Q																	✓				1
The ogilvie grading scale														✓							1
DIS				±												±			±		0 (3)
SF-36		±														±					0 (2)
SF-12		±														±					0 (2)
SF-6D		±														±					0 (2)

*RDQ* The reflux disease questionnaire, *QOLRAD* Quality of life in reflux and dyspepsia, *GERD-HRQL* The gastroesophageal reflux disease-health-related quality of life, *EQ-5D* EuroQOL-5D, *GERD-Q* Gastroesophageal reflux disease-questionnaire, *EORTC-QLQ-OES18* The European Organization for Research and Treatment of Cancer-Quality of life questionnaire-Oesophageal cancer module, *EORTC-QLQ-C30* The European Organization for Research and Treatment of Cancer-Quality of life questionnaire, *HADS* Hospital anxiety and depression scale, *SF-16* The 16-item short form health survey questionnaire, *SF-12* The 12-item short form health survey questionnaire, *SF-6D* Short form-6 dimension, *PROMIS-10* Patient-reported outcomes measurement information systems, *LASA* Linear analog self-assessment, *WHOQOL-BREF* World health organization quality of life instruments, *EORTC-QLU-C10D* European Organization for Research and Treatment of Cancer-Quality of life questionnaire-Core 10, *BSI-18* Brief symptom inventory, *GSRS* Gastrointestinal symptom rating scale, *GIQLI* Gastrointestinal quality of life index, *SCL-90* The symptom checklist-90-revised, *QLQ-OG25* EORTC quality of life questionnaire-Oesophago-gastric module, *TPS* Trust in physician scale, *IES* The impact of event scale, *DIS* The discomfort intolerance scale, PSQI, *Berlin-Q* Berlin Questionnaire, B-IPQ: Brief-Illness perception scale, CWS: Cancer worry scale, WOCS: Worry of cancer scale

✓ Factor was directly addressed,  ± factor was indirectly addressed

The cancer-specific PROMs (EORTC-QLQ C30, EORTC-QLQ C10D) and the generic WHOQOL-BREF measured items of sleeping difficulties in addition to anxiety and pain and indirectly addressed the burden of the use of medication.

Looking at more disease-specific measures, we found that the GIQLI, GERD-HRQL covered all factors related to GI symptoms. Furthermore, the GERD-HRQL addressed an item on lifestyle, whereas the GIQLI contained an item on support of family.

The EORTC-QLQ-OES18 was the only PROM with items on diet and lifestyle; this factor was only indirectly addressed by the GERD-HRQL and the QOLRAD. The other cancer-specific PROM, the QLQ-OG25, addressed GI symptoms, as well as anxiety and worry. The factors ‘sense of control’ and ‘knowledge and understanding’ were measured by items of the B-IPQ. Although fear of cancer was stated as an important factor influencing HRQoL in the literature, it was only measured in one study using the CWS [38]. In another study by Rosmolen et al. [21, 22], the WOCS was used for assessing fear of cancer (recurrence). However, we found no accurate validation in the references.

The TPS was the only PROM measuring ‘trust in the physician.’ The factors uncertainty (QOLRAD) and endoscopic burden (IES) were only indirectly assessed. No PROMs with items on measuring the factor endoscopy as safety net were found. None of the studies address more than nine of the 18 factors important to patients with BE. Overall, a median of 7 (0–9) factors, stated as important to patients using validated PROMs, were covered.

## Discussion

In this systematic review, we identified 27 studies measuring HRQoL in BE patients; within these studies, 32 different PROMs were used. None of the identified PROMs were specifically validated to measure HRQoL in BE patients. Consequently, we found that a total of nine studies (33.3%) used some form of non-validated questionnaires. It is interesting to note that the total number of interventional studies that used HRQoL measurements is relatively low. These findings are in contrast with the increased number of endoscopic therapeutic options for BE patients resulting in publications [47].

The most frequently used PROMs for measuring generic HRQoL was the SF-36 (52.2%). Symptoms related to BE were frequently (83.4%) measured by the EORTC-QLQ-OES18, GERD-Q, GERD-HRQOL, QOLRAD, and the RDQ. The HADS was used to measure symptoms of anxiety and depression in 26% of studies.

We identified four studies with a qualitative design exploring factors influencing HRQoL according to BE patients. Within these studies, the following factors were addressed, namely fear of cancer, anxiety, trust in physician, sense of control, uncertainty, worry, burden of endoscopy, knowledge and understanding, GI symptoms, sleeping difficulties, diet and lifestyle, use of medication, and support of family and friends. These findings are fairly in line with those of Britton et al. [8]. In this study, symptom control, psychological effects as anxiety and depression, worry of cancer, patients’ subjective perceived risk of cancer, frequency and severity of worry, and disease-specific knowledge were considered key factors for assessing HRQoL in BE patients.

None of the studies addressed more than nine of the 18 factors important to patients with BE. Disease-specific PROMs were more successful in covering factors important to BE patients, compared to generic PROMs. Interestingly, generic PROMs were used in 77.8% of all studies. However, generic PROMs are used to provide comparisons between diseases or to compare data with population normative values, not to evaluate specific patient populations. The selection of PROMs is a complex but essential process. Several documents for guidance in the appropriate selection of PROMs in clinical trials are available [48]. The current review confirms the need of a more patient-centered approach in measuring HRQoL in BE patients. Since there is no BE-specific PROM available, the development of a new instrument seems inevitable. However, a wide variety of PROMs is currently available, and the development of a new measurement tool is time-consuming and complex. A combination of the following disease-specific PROMs GIQLI or GERD-HRQOL, with the CWS, TPS, the B-IPQ would be appropriate to measure factors influencing HRQoL in BE patients. This would, however, necessitate a large number of questions to be addressed by patients. Using the “Patient-Reported Outcomes Measurement Information System” (PROMIS) databank may be an appropriate solution for this problem. PROMIS is an easily accessible set of person-centered measures, using computerized adaptive testing from large item banks for over 70 domains relevant to a wide variety of chronic diseases [49–51]. PROMIS enables comparisons across populations and studies and can be integrated in several electronic health records. We advise clinicians to use the items: PROMIS® GI (disrupted and swallowing, reflux and gas and bloating), PROMIS® Anxiety, and PROMIS® Self-Efficacy (Managing medications and treatment, Managing Symptoms). Further research is needed to validate the PROMIS databank in BE patients.

The current study has some limitations that need to be addressed. First, the aim of this review was to identify studies that measure HRQoL in BE patients. Using MeSH and free-text words focusing on areas of HRQoL, we may have underestimated the number of interventional studies that used HRQoL as a secondary endpoint. Second, we identified only four studies with a qualitative study design. Of these, two studies directly investigated factors important to BE patients, while the other two used an indirect manner by focusing on patients experiences with surveillance endoscopy and patient burden, care delivery experience, and follow-up needs. However, all factors identified in the latter two studies were confirmed in the first two studies. Third, the list of factors important to BE patients and the degree to which factors were addressed by the various PROMs is subjective. To increase the intra-rater and inter-rater reliability, an independent extraction of potential factors was performed by two researchers.

In conclusion, none of the studies measuring HRQoL in BE patients sufficiently reflected the perceptions of HRQoL in BE patients. For the selection of PROMs, we encourage physicians and researchers measuring HRQoL to choose their PRO from a patient perspective and not strictly based on relevance according to health professionals’ definitions. Using PROMs that are more patient-centered will enhance knowledge of the true impact of surveillance and endoscopic treatment on the (perceived) functioning of BE patients.

## Data Availability

The data that support the findings of this study are available from the corresponding author upon reasonable request.

## Code availability

The primary methodology and inclusion criteria were published in a protocol in the PROSPERO database under registration number CRD42021224231.
